# The Role of Speech and Language Therapists (SLTs) in International Stroke Teams: A Systematic Review

**DOI:** 10.1111/1460-6984.70062

**Published:** 2025-06-19

**Authors:** Marina Charalambous, John E. Pierce, Georgia Pastou, Erasmia Kola, Sean I. Savitz

**Affiliations:** ^1^ Department of Rehabilitation Sciences, School of Health Sciences Cyprus University of Technology Limassol Cyprus; ^2^ Centre of Research Excellence in Aphasia Recovery and Rehabilitation La Trobe University Melbourne Victoria Australia; ^3^ Bibliographic Support and Collection Development Office Manager, Librarian of the School of Health Sciences Cyprus University of Technology Limassol Cyprus; ^4^ Institute for Stroke and Cerebrovascular Disease The University of Texas Health Science Center at Houston (UTHealth) Houston Texas USA

**Keywords:** countries, multidisciplinary team, rehabilitation, speech and language therapists, stroke, systematic review

## Abstract

**Background:**

Current published guidelines suggest that speech and language therapists (SLTs) should be part of stroke teams, but their involvement and roles according to country income are unknown.

**Aims:**

This review aims to (1) investigate the level of involvement of SLTs in acute stroke management, rehabilitation, and long‐term care, and (2) examine whether the roles and contributions of SLTs in stroke care vary according to a country's income level.

**Methods:**

A systematic review methodology was conducted by an expert librarian and three independent researchers based on the Preferred Reporting Items for Systematic Reviews and Meta‐Analyses (PRISMA) guidelines. This systematic review was registered on the PROSPERO website. The search strategy involved using MESH Terms ‘speech and language therapy’ AND stroke AND team* across six databases as follows: MEDLINE Complete, APA PsycInfo, CINAHL Plus, PubMed, Embase, and Scopus. The review was conducted using the Covidence software.

**Results:**

Out of 1142 titles identified, 42 studies met the criteria: 34 studies (80%) were from high‐income countries, five (12%) were from upper–middle‐income countries, and three (8%) were from low–middle‐income countries. No studies were published in low‐income countries.

**Conclusion:**

Lack of SLTs reported in stroke and rehabilitation teams in low‐ and low–middle‐income countries raises concern for patients' access to specialized SLT services. Healthcare policy should highlight the need for greater investment in SLT resources and the role of SLTs in managing aphasia, dysphagia, and chronic rehabilitation needs to improve patient outcomes.

**WHAT THIS PAPER ADDS:**

**What is already known on the subject**

- Stroke is a leading cause of acquired disability with rehabilitation requiring a multidisciplinary approach. Speech and language therapists (SLTs) play a significant role in stroke care, addressing communication, swallowing, and cognitive disorders. Despite the recognized importance, SLTs’ involvement in stroke care teams varies significantly across healthcare systems, especially in lower‐income countries.

**What this paper adds to existing knowledge**

- This study reveals global discrepancies in SLT involvement in stroke care, with high‐income countries benefiting from structured, specialized roles across all phases of rehabilitation, while low‐ and middle‐income countries face ad hoc, limited involvement due to systemic changes. The findings highlight the need for standardized protocols and greater investment in SLT resources to ensure equitable stroke rehabilitation services worldwide.

**What are the potential or actual clinical implications of this work?**

- The clinical implications of this study are as follows: (a) integrating SLTs in stroke teams, especially in underrepresented regions, is essential to improve rehabilitation outcomes, (b) advocacy efforts prioritize equal access to SLT services globally, supported by policy changes and education and (c) participatory research must involve patients as equal partners to address community‐specific rehabilitation priorities as involving stroke survivors and caregivers in the design of rehabilitation services has been shown to identify local barriers to access and shape more culturally appropriate interventions.

## 1 Introduction

Stroke is the second leading cause of death worldwide and the third cause of acquired disability in adults (Feigin et al. 2023). Rehabilitation after a stroke ideally involves a multidisciplinary (MDT) approach where different healthcare professionals work together to address the wide needs of people with stroke (Adeniji et al. 2023; Bernhardt et al. 2023). Among these professionals, speech and language therapists (SLTs) have a unique and essential role within the stroke team, providing interventions to address the communication, cognitive, and swallowing disorders that often arise after a stroke. These impairments can significantly affect the quality of life of stroke survivors, limiting their ability to engage in everyday activities, socialize, and maintain independence (Charalambous et al. 2020). Therefore, the timely and effective intervention of SLTs is vital for the functional recovery of people with stroke.

True MDT working goes beyond professionals delivering parallel interventions. In effective stroke care teams, SLTs collaborate closely with other professionals through shared goal setting, reciprocal referrals, and integrated therapy planning (Gopaul et al. 2023). For example, SLTs may guide physiotherapists in selecting communication‐accessible strategies for motor rehabilitation (Carragher et al. 2021) or support nurses in managing safe oral intake protocols (Dziewas et al. 2021). This integrative approach enhances the quality and coherence of rehabilitation (Hunt et al. 2022). In chronic care, SLTs often co‐develop community reintegration strategies with occupational therapists and social workers, ensuring the continuity of patient‐centred goals across disciplines (Eriksson et al. 2022).

In high‐income countries (HICs), well‐established guidelines outline the importance of SLT collaboration with other healthcare professionals. The World Stroke Organization Global Stroke Services Guidelines and Action Plan (Lindsay et al. 2014) highlight that SLTs should be involved early in stroke care, working alongside neurologists, physiotherapists, occupational therapists, dietitians, and nurses to ensure comprehensive care. In contrast to HICs, where SLTs are often embedded in stroke teams through formal guidelines and interdisciplinary care pathways (e.g., Wolfe et al. 2000; Godecke et al. 2021), low‐ and middle‐income countries frequently show inconsistent or minimal SLT involvement. Studies from South Africa, India, and Vietnam report ad hoc participation, limited workforce capacity, and lack of standardized protocols (Blackwell and Littlejohns 2010; Salunkhe et al. 2024; Phan et al. 2022). This disparity reflects broader systemic inequities in stroke rehabilitation infrastructure and professional training across global contexts. Building on this, it is important to examine how these differences in SLT involvement across income levels impact rehabilitation phases and healthcare systems more broadly.

Despite the recognized importance of SLTs in stroke rehabilitation, there is growing concern that their involvement in stroke care teams is inconsistent, particularly across the phases of stroke recovery (hyperacute, acute, subacute, and chronic) and different healthcare systems worldwide (Brady et al. 2022). In some settings, SLTs are integral members of stroke teams, contributing to assessment, intervention and ongoing care (Brady et al. 2022). However, in other settings, particularly in lower‐income countries, SLTs may be underrepresented or even absent (Feigin et al. 2022). The absence of SLTs affects not only access to specialist assessment and therapy but also impairs the capacity of other MDT members, such as nurses, physiotherapists and physicians, who may lack professional training or have the skills and knowledge to effectively engage with patients who have communication impairments (Carragher et al. 2021). This discrepancy raises significant concerns about equity in healthcare, as the absence of SLTs can lead to suboptimal recovery for stroke patients, particularly in areas such as communication and swallowing function.

Given identified gaps in SLT services in lower‐income countries (Feigin et al. 2022), we undertook a systematic review to examine the involvement of SLTs in stroke teams, including acute management, rehabilitation, and long‐term care across different countries and healthcare systems. Understanding these dynamics is crucial for several reasons to identify best practices that could be adopted in settings where SLT services are currently lacking, to inform healthcare policy by highlighting the need for greater investment in SLT resources and to contribute to a more equitable distribution of healthcare resources, ensuring that all stroke patients have access to the comprehensive care they need for optimal recovery.

This review aims to (1) investigate the level of involvement of SLTs in acute stroke management, rehabilitation, and long‐term care, and (2) examine whether the roles and contributions of SLTs in stroke care vary according to a country's income level.

## 2 Methods

### 2.1 Design

A systematic review was selected to address the involvement of SLTs in stroke teams, to provide syntheses of the state of knowledge in this field, and to identify future priorities (Muka et al. 2020). This systematic review was registered on the International Prospective Register of Systematic Reviews (PROSPERO) ID: CRD42024501909 and was conducted and reported in alignment with The Preferred Reporting Items for Systematic Reviews and Meta‐Analyses (PRISMA) reporting guidelines (Page et al. 2021). The PRISMA Checklist was used to endorse better reporting of the methodology and to guide the conduct and reporting of this review (see Appendix 1). The systematic search was implemented based on the PICO model (Eriksen and Frandsen, 2018) as a framework to ensure that the pertinent components of the question were well‐defined (Eriksen and Frandsen, 2018). The PICO question was formulated as follows:

**P**opulation (P—SLTs in stroke teams),
**I**ntervention (I—SLTs interventions in stroke teams in all phases of stroke care),
**C**omparison (C—to explore the presence vs. the absence of SLTs in stroke teams), and
**O**utcome (O—propose the inclusion of SLTs in stroke teams across countries).

### 2.2 Search Strategy and Selection Criteria

The research questions and the search terms were developed by the researchers (authors M.C., G.P., and E.K.). The search terms were related to the focused population, the language, and the types of study design to include in the review. An expert librarian in health sciences (co‐author EK) conducted a literature search from December 2023 to March 2024. Search strings were based on MESH Terms. The string of keywords searched were as follows: ‘speech and language therapy’ or ‘speech and language pathology’ ‘speech therapist’ or ‘speech pathologist’ or slt or slp AND stroke or ‘cerebrovascular accident’ or cva or ‘cerebral vascular event’ or cve or ‘transient ischaemic attack’ or tia or ‘acute stroke’ AND ‘patient care team’ or ‘multidisciplinary team’ or ‘integrative team’ or ‘interdisciplinary team’ or ‘interprofessional team’ or ‘healthcare team’ or team*. Six databases were selected for the review as follows: MEDLINE Complete, APA PsycInfo, CINAHL Plus, PubMed, Embase, and Scopus.

To be eligible for inclusion studies, should include (1) SLTs working with the adult population in stroke teams; (2) from any geographical location; (3) published in the English language and (4) include patients with a confirmed diagnosis of stroke. Studies involving (1) paediatric stroke, and (2) multiple patient populations (e.g., dementia, brain injury, cancer) were excluded.

### 2.3 Data Extraction and Synthesis

We used the Covidence systematic review software, Veritas Health Innovation, Melbourne, Australia, to screen and extract data. The two reviewers (first author MC and co‐author GP) identified studies that potentially fit the a priori determined criteria. We defined level of SLT involvement as: (a) full involvement where SLTs were embedded in multidisciplinary teams with shared care responsibilities; (b) partial involvement where SLTs were consulted for specific tasks but not involved in broader team processes; or (c) absent not mentioned as part of the stroke care team. This categorization aligns with approaches used in previous workforce mapping and service integration reviews (e.g., Owolabi et al. 2021; Strasser et al. 2005). We reviewed the titles and abstracts of studies retrieved through the search strategy. The abstracts of the selected studies were retrieved and assessed for eligibility by the two reviewers (MC and GP), who remained blinded during the process. Conflicts were resolved by the third reviewer (co‐author JEP), who was blinded to previous decisions. The full text of the selected abstracts was retrieved and assessed for eligibility by the two reviewers (MC and GP). Conflicts were resolved with a discussion between the two reviewers (MC and GP) until a consensus was reached.

The data extracted in response to the research questions were presented in tables including (1) the name of the authors and the year of publication, (2) the country where the research was conducted, (3) the phase of stroke care, (hyperacute, acute, subacute and chronic), (4) the setting, (5) the synthesis of the stroke team and (6) the role of the SLT.

### 2.4 Study Quality

The methodological quality of the included studies was assessed using the criteria outlined in the Cochrane Handbook for Systematic Reviews (Higgins et al. 2022). Discrepancies were resolved through discussion and consensus between the reviewers.

## 3 Results

**FIGURE 1:**
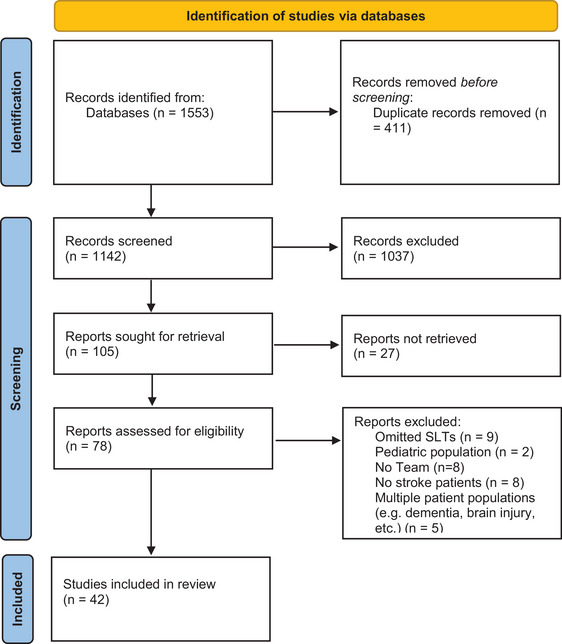
PRISMA 2020 flow diagram for the review process.

The initial search generated 1553 titles. Of these, 411 duplicates were removed, and 1037 citations were excluded by title and abstract. A total of 78 studies were identified for full‐text review, and after removal of 36 studies, 42 studies met the inclusion criteria. Please see Figure 1 for the flowchart of the process following the PRISMA guidelines.

Overall, most studies included in the review were observational in design, with relatively few randomized controlled trials (RCTs). The risk of bias varied across studies, with limitations often related to sample size, blinding, or unclear reporting of outcome measures. Supplementary table (Appendix 2) provides a detailed summary of the quality appraisal.

A total of 42 full‐text articles were included. Three tables were created to categorize the studies based on country income: low‐middle income (LMICs), upper‐middle income (UMICs), and high income (HICs) (World Development Indicators database, World Bank 2024). The data extracted from each of the articles are summarized in the three tables below: 34 papers published in HICs were identified (see Table 1), five were from UMICs (see Table 2), and three were from LMICs (see Table 3).

**TABLE 1:** Summary of the 34 published studies from high‐income countries in chronological order.

#	Author and year of publication	Country	Phase of stroke care	Setting	Team members		Role of the SLT
1.	Hinds and Wiles (1998)	United Kingdom	Acute	Hospital	Neurologist, SLT		Ax and Tx of swallowing disorders
2.	Holmqvist et al. (1998)	Sweden	Acute, subacute	Home‐based Rehab	PT, OT, SLT		Aphasia and communication Ax and Tx
3.	Lucas and Rodgers (1998)	United Kingdom	Acute, subacute	Hospital	Physicians, nurses, PT, SLT dieticians		Dysphagia Ax and Tx
4.	Kalra et al. (2000)	United Kingdom	Acute, subacute	Stroke unit + domiciliary care/outpatient setting	Stroke physician, PT, OT, SLT nurse		Speech and language Ax and Tx
5.	Wolfe et al. (2000)	United Kingdom	Acute, subacute	Home‐based community Rehab	PT, OT, SLT, community nurse	Speech, language, communication, swallow and cognitive Ax and Tx	
7.	von Koch et al. (2000)	Spain	Acute, subacute, chronic	Home rehabilitation	PT, OT, SLT	Speech, language and communication Ax and Tx	
8.	Pollack and Disler (2002)	Australia	Acute, subacute, chronic	Acute hospital, specialized rehab units and the community‐based rehab	PT, OT, SLT, nurses, neuroΨ, rehabilitation physician, social worker	Αx and Tx of communication, motor speech production, eating and swallowing difficulties	
9.	Huhmann et al. (2004)	United States of America	Acute	Hospital	Dietician, SLT, physicians	Bedside dysphagia Ax and Tx	
10.	Strasser et al. (2005)	United States of America	Acute, subacute	Hospitals	Physicians, nurses, PT, OT, SLT, social workers	Communication and cognitive skills Ax	
11.	Dey et al. (2005)	United Kingdom	Acute	Hospital	PT, SLT, nurses, physicians	Ax of swallowing and communication	
12.	Mohammed et al. (2006)	United Kingdom	Hyperacute, acute	Acute hospital with stroke unit	SLT, PT	Early screening/Ax of swallowing	
13.	Tan et al. (2007)	Ireland	Acute	Hospital	PT, SLT, OT, physicians, nutritionist	Swallow, speech and communication Ax	
14.	Warnecke et al. (2009)	Germany	Acute	Hospital	Neurologists, SLT, nursing staff, physicians	Dysphagia Ax (FEES)	
15.	Ringelstein et al. (2009)	Germany and Austria	Acute	Hospital stroke units	Emergency department staff, neurologist, stroke‐trained physician, diagnostic radiologist, cardiologist, internist, stroke‐trained nurses, social worker, SLT, PT	Early speech, language and swallow Ax and Tx	
16.	Ickenstein et al. (2012)	Germany	Acute	Stroke unit	Nursing staff, SLT, physicians	Clinical swallow Ax	
17.	Kaizer et al. (2012)	Canada	Acute	Rehab hospital	Dietician, nurse, PT, OT, SLT, Ψ, physician, pharmacist, respiratory therapist, social worker	Dysphagia Ax and Tx	
18.	Flamand‐Roze et al. (2012)	France	Acute	Stroke unit	Physicians, nurses, SLT	Speech, language and swallow Ax	
19.	O'Sullivan et al. (2014)	Ireland	Acute	Acute stroke centre	PT, OT, SLT, dietitians	Swallow and communication Ax and Tx	
20.	Hall et al. (2016)	Ireland	Subacute, chronic	Hospital	PT, OT, SLT	Language and swallow Ax and Tx	
21	Morrell et al. (2017)	United States of America	Acute	Urban‐certified stroke centres	SLT, neurologist, nurse	Dysphagia Ax and Tx	
22	Rice et al. (2017)	United States of America	Acute	Hospital	PT, OT, SLT	Language and cognitive deficits Ax and Tx	
23	Jhaveri et al. (2017)	United States of America	Acute, subacute	Home‐based rehab	Pharmacist, PT, OT, SLT, rehabilitation physician, social worker, geriatrician	Swallow Ax, cognitive screening	
24.	Schwarz et al. (2018)	Australia	Acute	Hospital	SLT, nurses	Swallow Ax, identification of signs and risk factors of dysphagia	
25.	Obana et al. (2019)	Japan	Acute	University hospital	Dentists, dental hygienists, nurses, SLT	Dysphagia Ax and Tx	
26.	Nelson et al. (2020)	Canada	Chronic	Community hospital	Nursing staff, PT, OT, SLT, recreation therapists, rehabilitation assistants, social workers, volunteers	Aphasia Ax and Tx volunteers cover for speech therapists’ shortage by providing targeted Tx aimed at improving patients’ functional communication	
27.	Chang et al. (2021)	South Korea	Acute, subacute	University hospital + home‐based rehab	Rehabilitation physiatrist, PT, OT, SLT, social worker	Communication Ax and Tx	
28.	Gerreth et al. (2021)	Poland	Subacute	Rehab centre	Dentist, physiatrist, neuroΨ, OT, SLT	Ax and Tx of dysphagia, dysarthria and aphasia	
29.	Carragher et al. (2021	Australia	Acute, subacute	Inpatient stroke rehab settings	PT, OT, SLT, nurses, dietician, nurses, medics	Dysphagia and communication Tx	
29.	Godecke et al. (2021)	Australia	Acute, subacute	Hospital	SLT, OT dietician, nurse, orthodontist	Acute: Dysphagia and communication Ax and Tx subacute: Improve the communication between allied health and patients	
30.	Nakamori et al. (2021)	Japan	Acute	Hospital	Doctors, nurses, SLT, dietitians	Swallow Ax	
31.	Eriksson et al. (2022)	Sweden	Chronic	Home‐based rehab	PT, OT, SLT, nurses, medical social workers, physicians, dietitians, assistant nurses	Member of an inter‐professional team for the rehabilitation of activities of daily living for people with stroke	
32.	Barnard et al. (2022)	United Kingdom	Hyperacute, acute	Hospital	Stroke physicians, SLT, SLT assistants, nurses, nurses’ assistants	Ax and Tx communication and swallowing disorders at the ward	
33.	Hunt et al. (2022)	Canada	N/A	Inpatient hospital‐based stroke rehab	PT, OT, SLT	Member of an inter‐professional rehabilitation team working on the cognitive orientation to daily occupational performance	
34.	Curtin et al. (2023)	Ireland	Hyperacute, acute	Tertiary referral hospital + stroke unit of university hospital	Geriatric medicine, dietitian, PT, OT, SLT, healthcare assistance, nurses, clinical neuroΨ	Support oral care before and/or after swallowing Ax	

Abbreviations: Ψ, psychologist; Ax, assessment; OT, occupational therapist; PT, physiotherapist; SLT, speech and language therapist; Tx, treatment.

**TABLE 2:** Summary of the five published studies from upper–middle‐income countries in chronological order.

#	Author and year of publication	Country	Phase of stroke care	Setting	Team members	Role of the SLT
1.	Blackwell and Littlejohns (2010)	South Africa	Acute, subacute	Private rehab clinics	Physicians, nursing staff, dietician, SLT, family members	Bedside Ax, clinical swallow Ax, modified barium swallow study, swallow and feeding Tx and rehabilitation
2.	Rodríguez‐Mutuberría et al. (2011)	Cuba	Acute, subacute	Rehab centre	PT, OT, SLT, Ψ, physiatrists, clinicians, nurses, neurologist	Ax and Tx of dysarthria, dysphagia and aphasia
3.	Anderle et al. (2019)	Brazil	Acute	Basic health units in primary care	SLT, PT, Ψ, nutritionists, neurologists,	Speech and language Ax and Tx, cognitive intervention, dysphagia and voice Ax and Tx
4.	Tay et al. (2023)	Malaysia	Chronic	Long‐term stroke care clinic	SLT, PT, dietician	Dysphagia Ax (videofluoroscopy)
5.	Wong et al. (2023)	China	Chronic	Outpatient community rehab centre	OT, PT, SLT	Aphasia and dysarthria Tx

Abbreviations: Ψ, psychologist; Ax, assessment; OT, occupational therapist; PT, physiotherapist; SLT, speech and language therapist; Tx, treatment.

**TABLE 3:** Summary of the three published studies from low–middle‐income countries in chronological order.

#	Author and year of publication	Country	Phase of stroke care	Setting	Team members	Role of the SLT
1.	Kanwal et al. (2022)	Pakistan	Acute	Stroke care units	PT, SLT, OT, Ψ, neurologist	Ax and Tx of communication disorders
2.	Phan et al. (2022)	Vietnam	Hyperacute	Emergency department and stroke units	Ψ/ neuroΨ, OT, SLT	Swallow Ax
3.	Salunkhe et al. (2024)	India	Acute	Hospital inpatient stroke care and rehab	OT, SLT, PT, neurologists, neurosurgeons	Swallow Ax, speech Tx

Abbreviations: Ψ, psychologist; Ax, assessment; OT, occupational therapist; PT, physiotherapist; SLT, speech and language therapist; Tx, treatment.

Across the 42 included studies, 26 described full SLT involvement in multidisciplinary stroke teams, 11 described partial involvement, and five reported no involvement. Full integration was predominantly observed in studies from HICs. The geographical distribution of studies shows that HICs dominated the literature, with 34 out of 42 studies (80%). Countries like the United Kingdom, Australia, and the United States had the most studies. The acute and subacute phases were the most frequently addressed in the published studies. Less coverage was evident in the chronic phase.

## 4 Discussion

### 4.1 What Is the Level of Involvement of SLTs in Stroke Teams?

Studies from HICs, particularly the United Kingdom (Barnard et al. 2022), Australia (Godecke et al. 2021), the United States (Jhaveri et al. 2017), Canada (Hunt et al. 2022), and Sweden (Holmqvist et al. 1998), indicate that SLTs are considered essential in multidisciplinary stroke teams in the acute phase. Similarly, evidence from Japan (Nakamori et al. 2021), Cuba (Rodríguez‐Mutuberría et al. 2011), South Africa (Blackwell and Littlejohns 2010), and India (Salunkhe et al. 2024) supports the inclusion of SLTs, although the role varies depending on the country's income level and healthcare infrastructure. The inclusion of SLTs was most common in acute and subacute rehabilitation, where they primarily collaborated with physiotherapists and occupational therapists. This collaboration was present across different countries.

In LMICs, their guidelines are less formalized about SLTs, and their role often depends on the availability of resources and trained professionals. For example, in South Africa, while SLTs are recognized as important team members, their involvement is limited by factors such as workforce shortages and uneven distribution of services (Blackwell and Littlejohns 2010). Similarly, in India, collaboration between SLTs and other professionals is often ad hoc, driven by local needs rather than standardized protocols (Salunkhe et al. 2024). In contrast, in LMICs, while the importance of such collaboration is acknowledged, it is often constrained by systemic challenges and their involvement appears quite limited in acute stroke care.

HICs, such as Ireland (Hall et al. 2016), the United Kingdom (Wolfe et al. 2000), and Sweden (Eriksson et al. 2022) provide evidence of SLTs' involvement in home‐based and community rehabilitation in the chronic phase of stroke. Specifically, SLTs play a pivotal role in setting patient‐centred goals in collaboration with stroke survivors and their caregivers, contributing to community re‐integration and psychosocial support (Schwarz et al. 2018) and better functional outcomes in the chronic phase (Hunt et al. 2022). However, in LMICs, the involvement of SLTs in the chronic phase of rehabilitation is less standardized. While SLTs are present in stroke teams in countries like South Africa (Blackwell and Littlejohns 2010) and India (Salunkhe et al. 2024), their roles are often limited due to resource constraints and a lack of trained professionals.

### 4.2 How Do the Roles and Contributions of SLTs in Stroke Care Vary?

The literature review reveals significant differences in the roles and contributions of SLTs in stroke care and rehabilitation, based on a country's income level. In HICs, SLTs focus on managing dysphagia (Labeit et al. 2023) and aphasia (Brady et al. 2022). Their involvement spans from screenings to formal assessments to long‐term rehabilitation, addressing both communication and swallowing disorders through advanced methods, for example, Fiberoptic Endoscopic Evaluation of Swallowing (FEES) for dysphagia assessment (Green et al. 2014), and well‐resourced rehabilitation programs, for example, ASPIRE‐S (Hall et al. 2016).

In UMICs, however, the focus of SLTs tends to be narrower, primarily addressing dysphagia and language disorders in the acute and subacute phases. In countries like South Africa, Cuba, Brazil, Malaysia and China, their roles were not as deeply integrated into stroke care.

In LMICs, SLTs' roles are often limited to general language needs rather than specialized stroke rehabilitation. For instance, in Vietnam, there was a shortage of SLTs and poor recruitment of allied health professionals in general hospitals (Phan et al. 2022). In countries like Pakistan and India, stroke units often do not have SLTs as evident from the published studies (Kanwal et al. 2022; Salunkhe et al. 2024).

## 5 Main Contribution

Our review highlights the need to integrate SLTs into stroke care teams, particularly in regions where they are under‐reported. Increased recognition, education, and healthcare policy changes are crucial for improving rehabilitation and patient outcomes. Advocacy should focus on equal access to SLT services globally. Health‐related participatory research should ensure that patients are equitable partners and work collaboratively to address community‐specific rehabilitation priorities.

## 6 Recommendations for Future Research

A future study could explore the impact of including SLTs in stroke teams on patient outcomes, by comparing rehabilitation effectiveness in teams with and without SLTs.

## 7 Limitations

Our study has limitations since we did not perform a direct analysis of health service teams. The relatively small number of studies published in LMICs and UMICs makes it difficult to profile the involvement of SLTs in stroke care and rehabilitation teams in these regions. We did not search articles without mention of SLTs; thus, it is unclear whether fewer resulting studies were due to the SLT not being included or fewer published papers overall. However, our findings show significant variations in SLT roles across different countries. Additionally, restricting the review to English‐language publications may have excluded relevant studies from non‐Anglophone LMICs, such as those in Francophone or Lusophone Africa and parts of Central Asia. Future reviews should consider including studies published in other languages to improve inclusivity and global representation.

## 8 Conclusion

The findings highlight significant disparities in the reported roles and contributions of SLTs in stroke care based on a country's income level. In LMICs, the minimal presence of SLTs underscores the urgency of investing in workforce development, recruitment, and retention. However, it is unclear whether the absence of SLTs in LMICs reflects a true absence of services or underrepresentation in the literature due to limited reporting or academic output. Standardizing stroke care guidelines across all income levels is critical to ensure that SLTs are fully integrated into multidisciplinary teams, enabling comprehensive rehabilitation services. UMICs demonstrate some SLT involvement, but greater emphasis is needed on enhancing multidisciplinary collaboration and expanding their roles in line with global best practices. Advocacy efforts should also focus on raising awareness of the importance of SLTs in managing dysphagia, aphasia, and other chronic rehabilitation needs, particularly in LMICs, to improve patient outcomes and quality of life.

## Conflicts of Interest

The authors declare no conflicts of interest.

## Supporting information




**Supplementary Table**: Methodological Quality of Included Studies.

## Data Availability

The data generated during the current study and support the conclusions of this article are publicly available in the manuscript. Any further data queries and requests should be submitted to the corresponding author, Marina Charalambous PhD, for consideration.

## Appendix 1

### The PRISMA 2020 Checklist

**Table:**

Section and Topic	Item #	Checklist item	Location where item is reported
**TITLE**			
Title	1	Identify the report as a systematic review.	1
**ABSTRACT**			
Abstract	2	See the PRISMA 2020 for Abstracts checklist.	1
**INTRODUCTION**			
Rationale	3	Describe the rationale for the review in the context of existing knowledge.	2
Objectives	4	Provide an explicit statement of the objective(s) or question(s) the review addresses.	3
**METHODS**			
Eligibility criteria	5	Specify the inclusion and exclusion criteria for the review and how studies were grouped for the syntheses.	3
Information sources	6	Specify all databases, registers, websites, organisations, reference lists and other sources searched or consulted to identify studies. Specify the date when each source was last searched or consulted.	3
Search strategy	7	Present the full search strategies for all databases, registers and websites, including any filters and limits used.	3
Selection process	8	Specify the methods used to decide whether a study met the inclusion criteria of the review, including how many reviewers screened each record and each report retrieved, whether they worked independently, and if applicable, details of automation tools used in the process.	3
Data collection process	9	Specify the methods used to collect data from reports, including how many reviewers collected data from each report, whether they worked independently, any processes for obtaining or confirming data from study investigators, and if applicable, details of automation tools used in the process.	3
Data items	10a	List and define all outcomes for which data were sought. Specify whether all results that were compatible with each outcome domain in each study were sought (e.g. for all measures, time points, analyses), and if not, the methods used to decide which results to collect.	4‐7
	10b	List and define all other variables for which data were sought (e.g. participant and intervention characteristics, funding sources). Describe any assumptions made about any missing or unclear information.	4‐7
Study risk of bias assessment	11	Specify the methods used to assess risk of bias in the included studies, including details of the tool(s) used, how many reviewers assessed each study and whether they worked independently, and if applicable, details of automation tools used in the process.	3
Effect measures	12	Specify for each outcome the effect measure(s) (e.g. risk ratio, mean difference) used in the synthesis or presentation of results.	n/a
Synthesis methods	13a	Describe the processes used to decide which studies were eligible for each synthesis (e.g. tabulating the study intervention characteristics and comparing against the planned groups for each synthesis (item #5)).	4‐7
	13b	Describe any methods required to prepare the data for presentation or synthesis, such as handling of missing summary statistics, or data conversions.	4‐7
	13c	Describe any methods used to tabulate or visually display results of individual studies and syntheses.	4‐7
	13d	Describe any methods used to synthesize results and provide a rationale for the choice(s). If meta‐analysis was performed, describe the model(s), method(s) to identify the presence and extent of statistical heterogeneity, and software package(s) used.	4‐7
	13e	Describe any methods used to explore possible causes of heterogeneity among study results (e.g. subgroup analysis, meta‐regression).	n/a
	13f	Describe any sensitivity analyses conducted to assess robustness of the synthesized results.	3
Reporting bias assessment	14	Describe any methods used to assess risk of bias due to missing results in a synthesis (arising from reporting biases).	3
Certainty assessment	15	Describe any methods used to assess certainty (or confidence) in the body of evidence for an outcome.	3
**RESULTS**			
Study selection	16a	Describe the results of the search and selection process, from the number of records identified in the search to the number of studies included in the review, ideally using a flow diagram.	4
	16b	Cite studies that might appear to meet the inclusion criteria, but which were excluded, and explain why they were excluded.	n/a
Study characteristics	17	Cite each included study and present its characteristics.	5‐7
Risk of bias in studies	18	Present assessments of risk of bias for each included study.	6
Results of individual studies	19	For all outcomes, present, for each study: (a) summary statistics for each group (where appropriate) and (b) an effect estimate and its precision (e.g. confidence/credible interval), ideally using structured tables or plots.	5‐7
Results of syntheses	20a	For each synthesis, briefly summarise the characteristics and risk of bias among contributing studies.	4‐7
	20b	Present results of all statistical syntheses conducted. If meta‐analysis was done, present for each the summary estimate and its precision (e.g. confidence/credible interval) and measures of statistical heterogeneity. If comparing groups, describe the direction of the effect.	n/a
	20c	Present results of all investigations of possible causes of heterogeneity among study results.	n/a
	20d	Present results of all sensitivity analyses conducted to assess the robustness of the synthesized results.	n/a
Reporting biases	21	Present assessments of risk of bias due to missing results (arising from reporting biases) for each synthesis assessed.	3
Certainty of evidence	22	Present assessments of certainty (or confidence) in the body of evidence for each outcome assessed.	3,4
**DISCUSSION**			
Discussion	23a	Provide a general interpretation of the results in the context of other evidence.	4,8
	23b	Discuss any limitations of the evidence included in the review.	8
	23c	Discuss any limitations of the review processes used.	8
	23d	Discuss implications of the results for practice, policy, and future research.	8
**OTHER INFORMATION**			
Registration and protocol	24a	Provide registration information for the review, including register name and registration number, or state that the review was not registered.	3
	24b	Indicate where the review protocol can be accessed, or state that a protocol was not prepared.	3
	24c	Describe and explain any amendments to information provided at registration or in the protocol.	8
Support	25	Describe sources of financial or non‐financial support for the review, and the role of the funders or sponsors in the review.	n/a
Competing interests	26	Declare any competing interests of review authors.	8
Availability of data, code and other materials	27	Report which of the following are publicly available and where they can be found: template data collection forms; data extracted from included studies; data used for all analyses; analytic code; any other materials used in the review.	8

From: Page MJ, McKenzie JE, Bossuyt PM, Boutron I, Hoffmann TC, Mulrow CD, et al. The PRISMA 2020 statement: an updated guideline for reporting systematic reviews. BMJ 2021;372:n71. https://doi.org/10.1136/bmj.n71. This work is licensed under CC BY 4.0. To view a copy of this license, visit https://creativecommons.org/licenses/by/4.0/.
