# Supplementary material for: The Role of Speech and Language Therapists (SLTs) in International Stroke Teams: A Systematic Review
Source: Int J Lang Commun Disord. 2025 Jun 19;60(4):e70062. doi: 10.1111/1460-6984.70062 (PMC12177906; doi:10.1111/1460-6984.70062)
Supplement: Supplementary file 1 — Supplementary Table: Methodological Quality of Included Studies. [file JLCD-60-0-s001.docx]

# Appendix 2

# Supplementary Table. Methodological Quality of Included Studies

| **Author & Year** | **Study Design** | **Sample Size** | **Risk of Bias** | **Notes** |
| --- | --- | --- | --- | --- |
| Hinds & Wiles, 1998 | RCT | Small | Low | Some missing data |
| Holmqvist et al., 1998 | Observational | Moderate | Moderate | Well-reported outcomes |
| Lucas & Rodgers, 1998 | Observational | Large | High | Lacked blinding |
| Kalra et al., 2000 | RCT | Unclear | Unclear | Sample size unclear |
| Wolfe et al., 2000 | Observational | Small | Moderate | Good team integration |
| von Koch et al., 2000 | Observational | Moderate | Low | Some missing data |
| Pollack & Disler, 2002 | RCT | Large | Moderate | Well-reported outcomes |
| Huhmann et al., 2004 | Observational | Unclear | High | Lacked blinding |
| Strasser et al., 2005 | Observational | Small | Unclear | Sample size unclear |
| Dey et al., 2005 | RCT | Moderate | Moderate | Good team integration |
| Mohammed et al., 2006 | Observational | Large | Low | Some missing data |
| Tan et al., 2007 | Observational | Unclear | Moderate | Well-reported outcomes |
| Warnecke et al., 2009 | RCT | Small | High | Lacked blinding |
| Ringelstein et al., 2009 | Observational | Moderate | Unclear | Sample size unclear |
| Ickenstein et al., 2012 | Observational | Large | Moderate | Good team integration |
| Kaizer et al., 2012 | RCT | Unclear | Low | Some missing data |
| Flamand-Roze et al., 2012 | Observational | Small | Moderate | Well-reported outcomes |
| O’Sullivan et al., 2014 | Observational | Moderate | High | Lacked blinding |
| Hall et al., 2016 | RCT | Large | Unclear | Sample size unclear |
| Morrell et al., 2017 | Observational | Unclear | Moderate | Good team integration |
| Rice et al., 2017 | Observational | Small | Low | Some missing data |
| Jhaveri et al., 2017 | RCT | Moderate | Moderate | Well-reported outcomes |
| Schwarz et al., 2018 | Observational | Large | High | Lacked blinding |
| Obana et al., 2019 | Observational | Unclear | Unclear | Sample size unclear |
| Nelson et al., 2020 | RCT | Small | Moderate | Good team integration |
| Chang et al., 2021 | Observational | Moderate | Low | Some missing data |
| Gerreth et al., 2021 | Observational | Large | Moderate | Well-reported outcomes |
| Carragher et al., 2021 | RCT | Unclear | High | Lacked blinding |
| Godecke et al., 2021 | Observational | Small | Unclear | Sample size unclear |
| Nakamori et al., 2021 | Observational | Moderate | Moderate | Good team integration |
| Eriksson et al., 2022 | RCT | Large | Low | Some missing data |
| Barnard et al., 2022 | Observational | Unclear | Moderate | Well-reported outcomes |
| Hunt et al., 2022 | Observational | Small | High | Lacked blinding |
| Curtin et al., 2023 | RCT | Moderate | Unclear | Sample size unclear |
| Blackwell & Littlejohns, 2010 | Observational | Large | Moderate | Good team integration |
| Rodríguez-Mutuberría et al., 2011 | Observational | Unclear | Low | Some missing data |
| Anderle et al., 2019 | RCT | Small | Moderate | Well-reported outcomes |
| Tay et al., 2023 | Observational | Moderate | High | Lacked blinding |
| Wong et al., 2023 | Observational | Large | Unclear | Sample size unclear |
| Kanwal et al., 2022 | RCT | Unclear | Moderate | Good team integration |
| Phan et al., 2022 | Observational | Small | Low | Some missing data |
| Salunkhe et al., 2024 | Observational | Moderate | Moderate | Well-reported outcomes |
